# Preoperative serum selenium predicts acute kidney injury after adult cardiac surgery

**DOI:** 10.1186/s12872-024-03825-y

**Published:** 2024-03-14

**Authors:** Guowei Fu, Shuying Bai

**Affiliations:** Department of Anesthesiology, Changzhou Second People’s Hospital, No.29, Xinglong Lane, Changzhou, 213003 China

**Keywords:** Selenium, Acute kidney injury, Cardiac surgery, Dose-response

## Abstract

**Background:**

The relationship between serum selenium (Se) and acute kidney injury after adult cardiac surgery (CSA-AKI) remains controversial. This study aimed to investigate the association of preoperative Se level with incident CSA-AKI.

**Method and Results:**

A retrospective cohort study was conducted on patients who underwent cardiac surgery. The primary outcome was incident CSA-AKI. Multivariable logistic regression models and natural cubic splines were used to estimate the association of Se levels and primary outcome. A total of 453 patient with a mean age of 62.97 years were included. Among all patients, 159 (35.1%) incident cases of CSA-AKI were identified. The level of preoperative Se concentration in patients with CSA-AKI was significant lower than that in patients without CSA-AKI. The higher preoperative Se level was significantly associated with decreased risk of CSA-AKI (adjusted OR 0.91, 95% CI: 0.87–0.99). Dose-response relationship curve revealed a nearly L-shape correlation between serum Se selenium levels and incident CSA-AKI.

**Conclusion:**

Our study suggested that a higher level of serum Se was significantly associated with lower risk of CSA-AKI. Further prospective studies are needed to clarify the causal relationship between serum Se level and incident CSA-AKI.

## Introduction

Acute kidney injury (AKI) is the most common major postoperative event of cardiac surgery, acting as a syndrome of sudden renal excretory dysfunction with high morbidity and mortality [1]. Cardiac surgery-associated acute kidney injury (CSA-AKI) ranked second among the causes of AKI in the intensive care setting [2], and mild serum creatinine increase after cardiac surgery indicated a higher morbidity, a longer length of hospital stay and increased healthcare costs [3]. Moreover, CSA-AKI is related to a long-term risk of death in patients with cardiac surgery independent of other risk factors, which even persisting in patients having complete renal recovery [4]. Given current varying AKI definitions and study population, the incidence of CSA-AKI ranged from 5% to as high as 42% [5]. A meta-analysis including 320,086 patients undergone cardiac surgery reported that the global incidence was 22.3% for all stages of CSA-AKI [6]. It’s therefore imperative to deploy prevention strategies for CAS-AKI by early identifying high-risk patients, ultimately improve the substantial health and socioeconomic burden.

In addition to female sex and advanced age, several comorbidities such as previous chronic kidney disease (CKD), diabetes mellitus, hypertension, cardiopulmonary diseases, and heart failure significantly increase the risk of CSA-AKI development [5]. The multifactorial mechanisms underlying the pathophysiology of CAS-AKI has been recognized but incompletely understood. In the setting of cardiac surgery, a variety of factors encompassing nephrotoxic drugs, hypotension, temporal renal ischemia, hemolysis, inflammation, and oxidative stress [7], may play roles in the development of AKI in different ways and to differing extents.

As an essential trace element, Selenium (Se) in the human body involves in lots of biological processes, especially antioxidant reactions [8]. The biological functions of Se are achieved by 25 selenoproteins with activating selenocysteine group [9]. The Se deficiency could impair human health and increase the risk of Keshan disease [10], cognitive impairment [11], and cardiovascular diseases [12]. The kidney plays an important role in maintain human selenium homeostasis by renal tubular epithelial cells absorbing Se and synthesizing GPx3 [13]. Animal study has shown that Se deficiency increases oxidative stress associated mitochondrial dysfunction which causes renal injury in mice [14]. Current literatures indicated potential clinical benefit of higher serum Se level in patients with chronic kidney disease [15, 16]. However, there is few data about Se level among patients who undergone cardiac surgery and knowledge gap continued to exist on the relationship between serum Se concentration and risk of CSA-AKI. Thus, this retrospective study aimed to investigate the association between serum Se and the incidence of CSA-AKI among population undergone cardiac surgery.

## Methods

### Study population

Between January 2011 and December 2018, we initially screened 629 patients aged more than 18 year-old who underwent cardiac surgery (including coronary artery bypass grafting [CABG] and open-heart valve repair or replacement surgery) at Changzhou Second People’s Hospital in China. For those having multiple cardiac surgeries, only the first one was selected. The exclusion criteria are as following: (1) patients with preoperative CKD 5 stage, i.e., an estimated glomerular filtration rate (eGFR) < 15 mL/min/1.73 m^2^, or undergoing maintenance dialysis, (2) with a prior AKI requiring dialysis within 1 year of index surgery, (3) died within 48 h after surgery, (4) with missing information on serum Se and creatinine levels. This single-center, retrospective, observational cohort study was approved by the local Institutional Review Board (No. 2022105-10) and was performed in adherence with the Declaration of Helsinki. The requirement for obtaining informed consent was waived because of the retrospective design.

### Primary outcome

The primary outcome of the current study was incident CSA-AKI. At our center, serum creatinine was regularly measured the day before surgery. The change from the maximal postoperative serum creatinine level within 7 days after surgery to the last baseline value was used to diagnosis CSA-AKI and determine its stage. The Kidney Disease Improving Global Outcomes (KDIGO) criteria [1] was adopted to define CAS-AKI: Stage 1 was defined as creatinine increase by 1.5–1.9 times baseline within 7 d or increase by ≥ 0.3 mg/dL within 48 h, Stage 2 as a 2-2.9 times baseline increase, and Stage 3 as ≥ 3 times baseline or increase to ≥ 4 mg/dL or dialysis.

### Se determination and study covariates

Demographic information, comorbidities, laboratory tests, and perioperative data were collected from medical health recording. Blood samples for Se determination were taken from patients after a 12 h fasting period preoperatively. The whole blood was centrifugated to obtain serum, which was immediately stored at -80℃ until analysis. A Spectra AA 220 Z (Varian) based on the carbon-furnace atomic-absorption spectrometry and Zeeman compensation was used to determine the serum Se concentrations. The extracted serum was firstly thawed and diluted with 5% nitric acid. The samples were further mixed homogeneously in a vortex mixer and then microwaved at 90 °C for 1 h in a microwave digester. We centrifuge and extract the supernatant for further evaluation at room temperature. The 1% solvent of diammonium hydrogen phosphate was used as matrix modifer in the experiment.

### Statistical analysis

The numerical data are presented as mean ± standard deviations (SD) if normally distributed or otherwise median with interquartile range (IQR), and groups comparisons were performed by the Mann-Whitney or Kruskal-Wallis test as indicated. The categorical data are summarized as count with frequencies and compared by the chi-square test. Apart from continuous form, preoperative serum Se levels were further categorized into quartiles to assess a stepwise association. Multivariable logistic regression models were used to estimate the association of Se levels and primary outcome by generated adjusted odds ratios (ORs) with 95% confidence intervals (CIs). The variables in multivariable models included baseline features showing statistical significance in the univariate analysis for CSA-AKI and those considered to have clinical significance [17]. The stratified analyses were conducted to explore the potential interaction effects between baseline variables and serum Se by adding a multiplicative interaction item in models. Natural cubic splines were adopted to explore the concentration-response curve between serum Se level and incident CSA-AKI events. All analysis will be performed on the complete cases due to low proportion of missing value. All statistical analyses were performed using R software, version 4.0.0 for Windows (R Foundation for Statistical Computing, Vienna, Austria, 2019), and 2-tailed *P* values less than 0.05 was considered statistically significant.

## Results

### Baseline characteristics and primary outcome

**Fig. 1:**
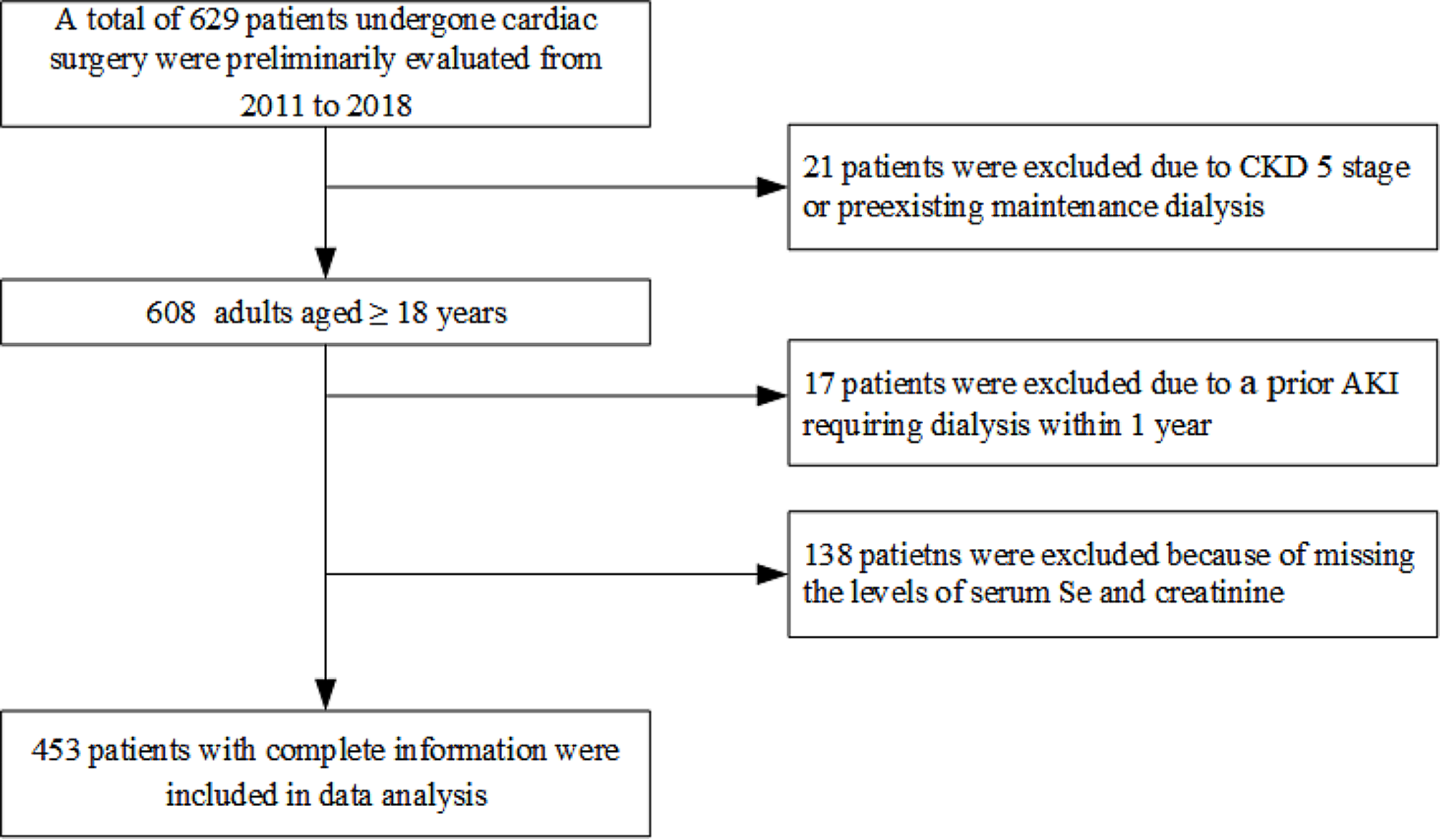
Study flowchart of the patients selection

Among initially screened 629 patients undergone cardiac surgery, a total of 453 patents fulfilling inclusion criteria were finally included after assessment (Fig. 1). Table 1 depicted the baseline characteristics of study patients. The 40.18% of patients were female, with a mean age of 62.97 ± 15.58 years. Regarding surgery type, 175 (38.63%) patients were treated by CABG, and the rest treated by valve surgery. The mean serum creatinine, hemoglobin, albumin, and Se before surgery was 86.79 ± 9.53 µmol/L, 127.7 ± 8.05 g/dL, 4.02 ± 0.95 g/dL, and 158.92 ± 6.72 µg/L, respectively.

**Table 1:** Baseline characteristics between patients with and without CSA-AKI

	Total (*n* = 453)	Non-AKI (*n* = 294)	AKI (*n* = 159)	*P* value
Age (years)	62.97 (15.58)	62 (15.84)	64.75 (14.96)	< 0.001
Female sex, n (%)	182 (40.18)	104 (35.37)	78 (49.06)	0.005
BMI (kg/m^2^)	26.85 (4.61)	26.31 (4.55)	27.85 (4.58)	< 0.001
Smoking, n (%)	92 (20.31)	63 (21.43)	29 (18.24)	0.421
Hypertension, n (%)	172 (37.97)	113 (38.44)	59 (37.11)	0.781
Diabetes, n (%)	122 (26.93)	77 (26.19)	45 (28.3)	0.629
Chronic heart failure, n (%)	149 (32.89)	53 (18.03)	96 (60.38)	< 0.001
Atrial fibrillation, n (%)	59 (13.02)	14 (13.46)	45 (57.69)	< 0.001
COPD, n (%)	9 (1.99)	5 (1.70)	4 (2.52)	0.553
Surgery type, n (%)				0.750
CABG	175 (38.63)	112 (38.1)	63 (39.62)	
Valve surgery	278 (61.37)	182 (61.9)	96 (60.38)	
Anesthesia time (min)	228.45 (40.52)	227.82 (39.23)	229.61 (42.9)	< 0.001
CPB time (min)	120.96 (27.33)	115.54 (24.47)	131 (29.49)	< 0.001
Serum creatinine (µmol/L)	86.79 (9.53)	85.79 (8.02)	88.63 (11.63)	< 0.001
Hemoglobin (g/dL)	127.7 (8.05)	129.16 (8.45)	125 (6.43)	< 0.001
Albumin (g/dL)	4.02 (0.95)	4.17 (0.82)	3.73 (1.08)	< 0.001
Preoperative Se (µg/L)	158.92 (6.72)	159.89 (5.7)	157.13 (8)	< 0.001

CSA-AKI, cardiac surgery-associated acute kidney injury; BMI, body mass index; COPD, chronic obstructive pulmonary disease; CABG, coronary artery bypass grafting; CPB, cardiopulmonary bypass; Se, selenium

Among all patients, 159 (35.1%) incident cases of CSA-AKI were identified. Of which, 126 (79.2%) were recorded as KDIGO 1 stage, versus 33 (20.8%) as KDIGO 2–3 stage. Compared to those without CSA-AKI, individuals with CSA-AKI were older and tend to have more comorbidities such as heart failure and atrial fibrillation. Moreover, patients with CSA-AKI exhibited a higher preoperative serum creatinine level, a longer anesthesia time and CPB time, lower level of hemoglobin and albumin. The level of preoperative Se concentration in patients with CSA-AKI was significant lower than that in patients with patients without CSA-AKI.

### Group differences according to serum Se level quartile

When patients were stratified by quartiles of serum Se level (Table 2), the lowest quartile group more often presented as older age, females, higher BMI, longer anesthesia time, longer CPB time, higher serum creatinine level, lower hemoglobin and albumin level.

**Table 2:** Baseline characteristics according to preoperative serum Se level quartile

	Q1 (154.46–163.30 µg/L)	Q2 (155.98-158.33 µg/L)	Q3 (160.25-162.33 µg/L)	Q4 (164.60-168.74 µg/L)	*P* value
No. of patients	114	113	113	113	
Age (years)	61.63 (16.52)	62.81 (16.81)	62.55 (14.74)	64.88 (14.07)	< 0.001
Female sex, n (%)	60 (52.63)	49 (43.36)	37 (32.74)	36 (31.86)	0.003
BMI (kg/m^2^)	27.65 (4.74)	26.8 (4.82)	26.69 (4.56)	26.24 (4.25)	< 0.001
Smoking, n (%)	20 (17.54)	18 (15.93)	28 (24.78)	26 (23.01)	0.286
Hypertension, n (%)	42 (36.84)	33 (29.2)	43 (38.05)	54 (47.79)	0.039
Diabetes, n (%)	28 (24.56)	33 (29.2)	28 (24.78)	33 (29.2)	0.757
Chronic heart failure, n (%)	46 (40.35)	38 (33.63)	31 (27.43)	34 (30.09)	0.185
Atrial fibrillation, n (%)	18 (30)	18 (36.73)	9 (24.32)	14 (38.89)	0.240
COPD, n (%)	2 (1.75)	2 (1.77)	2 (1.77)	3 (2.65)	0.951
Surgery type, n (%)					0.039
CABG	43 (37.72)	43 (38.05)	55 (48.67)	34 (30.09)	
Valve surgery	71 (62.28)	70 (61.95)	58 (51.33)	79 (69.91)	
Anesthesia time (min)	226.51 (38.79)	230.46 (43.48)	233.95 (42.24)	222.88 (36.94)	< 0.001
CPB time (min)	125.17 (27.67)	118.93 (26.17)	123.29 (26.89)	116.43 (28.02)	< 0.001
Serum creatinine (µmol/L)	88.9 (10.53)	85.65 (9.17)	85.49 (8.86)	87.1 (9.18)	< 0.001
Hemoglobin (g/dL)	126.37 (7.67)	128.58 (7.54)	127.82 (8.42)	128.05 (8.46)	< 0.001
Albumin (g/dL)	3.83 (0.94)	4.16 (0.98)	4.07 (0.95)	4 (0.89)	< 0.001

CSA-AKI, cardiac surgery-associated acute kidney injury; BMI, body mass index; COPD, chronic obstructive pulmonary disease; CABG, coronary artery bypass grafting; CPB, cardiopulmonary bypass; Se, selenium

### Associations between serum Se levels and CSA-AKI

**Fig. 2:**
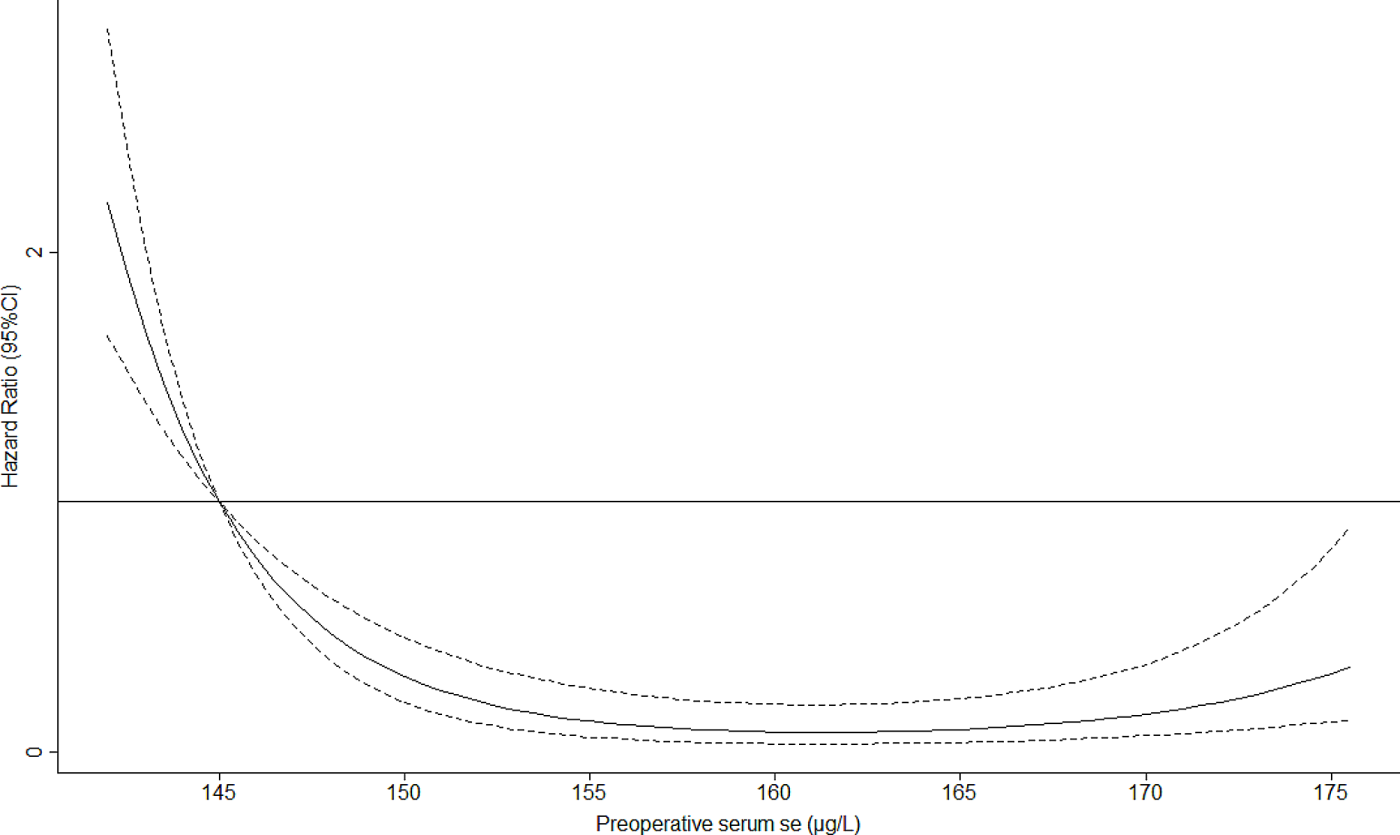
Natural cubic spline analysis between serum selenium concentrations and CSA-AKI

Table 3 presented the associations between preoperative Se levels and incident CSA-AKI from multivariate regression analyses. In multivariable analyses, we found that a higher preoperative Se level (adjusted OR 0.91, 95% CI: 0.87–0.99) was significantly associated with decreased risk of CSA-AKI, after adjusting age, sex, BMI, hypertension, diabetes, chronic heart failure, atrial fibrillation, CPB time, serum creatinine, hemoglobin and albumin. Similarly, in comparison with patients in the lowest quartile, those with the highest quartile were associated with lower risk of CSA-AKI [adjusted OR (95% CI): 0.17 (0.08–0.34)]. Dose-response relationship curve revealed a nearly L-shape correlation between serum Se selenium levels and incident CSA-AKI (Fig. 2). Subgroup analyses showed the similar findings with the main results across different subgroups (Table 4).

**Table 3:** Logistic regression analyses on clinical outcome for preoperative Se levels

	Univariate analyses		Multivariate analyses	
	OR (95% CI)	*P* value	OR (95% CI)	*P* value
Age (years)	1.01 (0.99–1.03)	0.073	1.00 (0.99–1.01)	0.783
Female sex, n (%)	1.76 (1.19–2.60)	0.005	1.26 (0.76–2.10)	0.375
BMI (kg/m^2^)	1.07 (1.03–1.12)	0.001	1.06 (1.00-1.11)	0.036
Smoking, n (%)	0.82 (0.50–1.33)	0.421		
Hypertension, n (%)	0.95 (0.63–1.41)	0.781	1.22 (0.72–2.05)	0.456
Diabetes, n (%)	1.11 (0.72–1.71)	0.629	1.11 (0.63–1.95)	0.723
Chronic heart failure, n (%)	6.93 (4.48–10.71)	< 0.001	4.30 (2.51–7.37)	< 0.001
Atrial fibrillation, n (%)	7.90 (4.17–14.94)	< 0.001	5.05 (2.35–10.85)	< 0.001
COPD, n (%)	1.49 (0.40–5.64)	0.555		
CABG vs. Valve surgery	1.07 (0.72–1.58)	0.750		
Anesthesia time (min)	1.00 (0.99–1.01)	0.653		
CPB time (min)	1.02 (1.01–1.03)	< 0.001	1.01 (1.00-1.02)	0.009
Serum creatinine (µmol/L)	1.03 (1.01–1.05)	0.003	1.04 (1.01–1.06)	0.008
Hemoglobin (g/dL)	0.93 (0.91–0.96)	< 0.001	0.93 (0.90–0.96)	< 0.001
Albumin (g/dL)	0.60 (0.48–0.75)	< 0.001	0.65 (0.49–0.85)	0.002
Preoperative Se (µg/L)				
Q1	Ref		Ref	
Q2	0.37 (0.21–0.64)	< 0.001	0.20 (0.10–0.40)	< 0.001
Q3	0.30 (0.17–0.52)	< 0.001	0.22 (0.11–0.43)	< 0.001
Q4	0.39 (0.22–0.67)	< 0.001	0.17 (0.08–0.34)	< 0.001
P for trend^*^		< 0.001		< 0.001
Per 1 unit	0.94 (0.91–0.97)	< 0.001	0.91 (0.87–0.99)	< 0.001

^*^P for trend: calculated by treating quartiles as a continuous variable in each model

**Table 4:** Subgroup analyses

	OR (95% CI)^*^	P for interaction
Age (years)		0.763
<65	0.93 (0.88–0.98)	
≥ 65	0.89 (0.84–0.94)	
Sex		0.305
Male	0.89 (0.84–0.94)	
Female	0.92 (0.87–0.97)	
Hypertension		0.803
Yes	0.89 (0.84–0.96)	
No	0.91 (0.86–0.95)	
Diabetes		0.567
Yes	0.92 (0.85–1.01)	
No	0.90 (0.84–0.94)	
Chronic heart failure		0.937
Yes	0.90 (0.84–0.96)	
No	0.91 (0.86–0.95)	
Atrial fibrillation		0.250
Yes	0.92 (0.85–1.01)	
No	0.90 (0.86–0.94)	

^*^Effect values are calculated by treating Se as a continuous variable

All covariates in Table 3 were adjusted in the model when they were not the strata variables

## Discussion

In this retrospective cohort study, we investigated the association between preoperative Se levels and CSA-AKI development amongst patients undergoing cardiac surgery. We observed a relatively high incidence of CSA-AKI in patents with low Se levels, and found that a higher serum Se level was significantly associated with decreased risk of incident CSA-AKI even after adjusting for confounding variables.

Benefiting from the increasing epidemiological knowledge, the role of Se (including organic or inorganic forms) has been related to a number of clinical diseases [18]. Different from observational findings, interestingly, current intervention studies with Se supplements in population all failed to prove the preventive effect of Se to reduce the risk of type 2 diabetes or cancers [19, 20]. These controversial results suggest the complexity pathophysiological effects of Se in vivo and potential threshold effects. Several studies have reported the associations between serum Se and morbidity and mortality of kidney disorders, mainly focusing on CKD. Patients with CKD are always characterized by active inflammation and oxidative stress, as well as abnormal metabolisms of microelements. Previous observational studies demonstrated that patients with CKD had a lower serum Se levels than those of healthy adults [16, 21]. Xie et al. found that Se intake seemed to have an inverse relationship on CKD development [15]. Zhu et al. analyzing 3,063 CKD adults from NHANES database reported that a higher serum selenium concentration could attenuate the risk of all-cause and CVD mortality in patients with CKD, albeit without estimated adequate dose recommendation [22].

In contrast, the evidence regarding the relationship between serum Se level or Se supplementation and CSA-AKI development after cardiac surgery is still scarce. A joint supplement intervention trial comprising Se supplements found that Se 600 mg twice a day did not reduce the risk of AKI in patients with elective off-pump CABG [23]. In our study, we firstly reported that a higher serum Se level could significantly reduce the risk of CSA-AKI. We conjecture that heterogeneity in study designs, including patients, serum Se levels, and surgery (CABG and valve surgery in our study) have contributed to differences between the two studies. Serum Se concentration largely varies among different population partially due to the differential geographical distribution of Se in soil. The main sources of dietary Se are meat and eggs, followed by grains such as flour and rice [24]. Se deficiency is common globally especially in China, while some countries such as Venezuela, Canada, the United States and Japan have high intakes of Se (> 100 µg/day) [25]. Considering potential differences in dietary habits, selenium distribution, and genetic background, a note of caution must be introduced about the generalizability of our findings to other populations.

Our findings supported the role of preoperative Se levels in identifying high-risk patients with CSA-AKI, and suggested that comprehensive perioperative management should be implemented to reduce the risk of AKI in patients with low serum Se levels or selenium deficiency. In this study, we also observed a nearly L-shaped correlation between serum Se levels and incident CSA-AKI. This finding is similar to previous studies focusing on populations with heart failure and CKD [22, 26], despite with different baseline serum Se concentration. As reported, however, the serum Se level associated with minimal mortality risk is 130–150 µg/L in the general population [27]. These results suggest that the health benefits of Se have a potential threshold effect, and only appropriate serum Se levels can exert organ protective benefits. Limited by highly selective population and sample size, our study failed to identify Se concentrations with the lowest risk of AKI. Additionally, we did not observe interactions between Se and age, sex, hypertension, diabetes, heart failure and atrial fibrillation for the AKI odds. Although diabetes status possibly affect the Se metabolism [28], consistently protective effects of the higher Se level existed in patients with or without diabetes. These preliminary evidence calling for future large-sample research to clarify the possible association between Se and prevention of CSA-AKI.

Regarding the definition of AKI, we adopt the diagnostic criteria of KIDGO rather than the Risk, Injury, Failure, Loss, End-Stage (RIFLE), or Acute Kidney Injury Network (AKIN) criteria. Compared to its predecessors (the RIFLE and AKIN scales), the KIDGO criteria has showed the greater sensitivity to identify earlier kidney injury and better ability of predicting mortality [29, 30]. Notably, The KDIGO scale is also based on the one-fits-all, single measurement of plasma creatinine concentration. This criteria failed to define those AKI not detected by plasma creatinine changing when undamaged nephrons provided recruitment of renal functional reserve [31]. Considering potential multi-etiopathological nature of AKI, future diagnostic algorithms which accounting for injury biomarkers, renal blood flow, and etiopathological features may help to accurately identify AKI.

Several underlying mechanisms of Se involvement in kidney health have been discussed. The most noteworthy aspect is the antioxidant effect of Se by acting as a cofactor of antioxidant GPx enzymes [9]. Se nanoparticles may alleviate AKI induced by ischemia reperfusion injury by upregulated the (GPx)-1 levels and suppressed NLRP3 inflammasome [32]. When selenoprotein expression are saturated, however, excessive serum Se will bind to nonspecific selenium-containing proteins and exert harmful effects through selenomethionine (SeMet) in place of methionine [33]. The metabolites of SeMet including selenols/selenates can product superoxide radicals and selenyl sulphides/disulphides, causing protein aggregation, endoplasmic reticulum stress, and inactivation of transcription factors [34]. This could to some extent explain the negative results in the Se supplementation trails [19, 20, 23]. In addition, Se is involved in the synthesis and activity of deiodinases, and then through regulating thyroid hormone influences renal hemodynamics [35]. Moreover, Se has been found related to cellular immunity and humoral immunity, and Se depletion could impaired lymphocytic proliferation, macrophage activation, cytokine generation, and neutrophil chemotaxis [36, 37].

### Limitations

Several limitations should be considered when interpreting our findings. First, like other observational studies, we cannot derive a causal relationship between Se level and AKI after cardiac surgery especially due to single center and small sample size. And the generalization of our findings should be with caution because of selective population. Second, residual confounders or unmeasured factors may interfere the results due to observational nature. However, multivariate analyses and subgroup analyses showed robust and consistent results. And we have further validated the step-wise associations by Se quartile and a dose-response curve, which lowers the chances of bias. Third, we exclude patients without Se measurement, which causing potential selection bias. Finally, we only assessed the serum Se concentration at baseline because of the design of a short-term exposure-outcome study. Future research should take the dynamic changes of Se concentration into account.

## Conclusion

In this study, preoperative higher serum Se level was significantly associated with lower risk of incident CSA-AKI in patients undergoing cardiac surgery. These findings suggested that serum Se maybe a modifiable risk factor for CSA-AKI and be used to identify patients at the high risk of CSA-AKI development. Further prospective multicenter studies are needed to clarify the causal relationship between serum Se level and incident CSA-AKI.

## Data Availability

The datasets used and analyzed during the present study are available from the corresponding author on reasonable request.
